# Innovative Treatment Approach: Multiple Variability (MV) Loop Intervention for Angle Class I Malocclusion with Dewey's Type 2 Modification: A Case Report

**DOI:** 10.7759/cureus.64733

**Published:** 2024-07-17

**Authors:** Narendra Sharma, Ranjit Kamble, Srushti Atole, Sumukh Nerurkar, Japneet Kaiser

**Affiliations:** 1 Department of Orthodontics and Dentofacial Orthopedics, Sharad Pawar Dental College and Hospital, Datta Meghe Institute of Higher Education and Research, Wardha, IND

**Keywords:** therapeutic premolar extraction, friction mechanics, frictionless mechanics, multiple variability loop, bimaxillary protrusion

## Abstract

Proclination of anteriors is significantly found in developing malocclusions and affecting both aesthetics and function. In patients with protrusions or crowding, extraction therapy is often necessary. Orthodontic treatment is initiated with the extraction of maxillary first premolars to address the protrusions or crowding and achieve a harmonious occlusion. There are two ways to retract anteriors during extraction space closure: friction or frictionless. The present case report explains the innovative treatment approach for the correction of anterior protrusion using multiple variability (MV) loops. Even with the good efficiency of the MV loop, meticulous wire bending is one of the disadvantages of the loop, and it requires clinician skills. Smaller loop fabrication will be the future scope of the appliance to increase patient compliance toward the treatment using MV loop as it will cause less hindrance in the vestibular region.

## Introduction

In order to address malocclusions, space closure is an essential component of orthodontic treatment. Based on the severity of the malocclusion, either the existing space or additional space created by the extraction of premolars can be utilized to treat a malocclusion [1]. Premolar extractions are widely approved as a treatment for malocclusion in patients who have severe crowding, proclination in both jaws, convex facial profiles, significant cephalometric disparity, and borderline instances [2]. In orthodontics, space closure is one of the most demanding procedures to perform. To minimize unfavorable side effects, a thorough understanding of biomechanics is necessary. Two types of mechanics are used to enable orthodontic tooth movement during space closure: frictional/sliding mechanics and frictionless/loop mechanics [3].

Frictionless mechanics is more beneficial as there is no force dissipation from friction during the retraction of anteriors. One of the main benefits of frictionless mechanics is that teeth receive a controlled force system. Simple vertical loops were the starting point of frictionless mechanics, which has progressed to increasingly complicated loop designs in order to improve the moment-to-force ratio and provide continuous light force. A loop with a low force-to-deflection rate will produce the desired tooth movement with a relatively small amount of force. For bodily movement, a moment-to-force (M/F) ratio of 10:1 is ideal for the loop. The M/F ratio can be optimally achieved by incorporating loop designs that enhance arch wire resiliency [3].

A multiple variability (MV) loop was developed by Jadhav et al. in 2020 [4]. It is recommended that titanium molybdenum alloy (TMA) wire, measuring 0.019 by 0.025 inches, be used for MV loop preparation to ensure proper utilization. In this way, the loop's capabilities will be maximized, and its performance will be improved in terms of moving teeth [4]. This case report explains how to correct a Class I malocclusion with Dewey’s type 2 modification (class I malocclusion with protrusive maxillary incisors - given by Martin Dewey in 1915) using an MV loop.

## Case presentation

**Figure 1:**
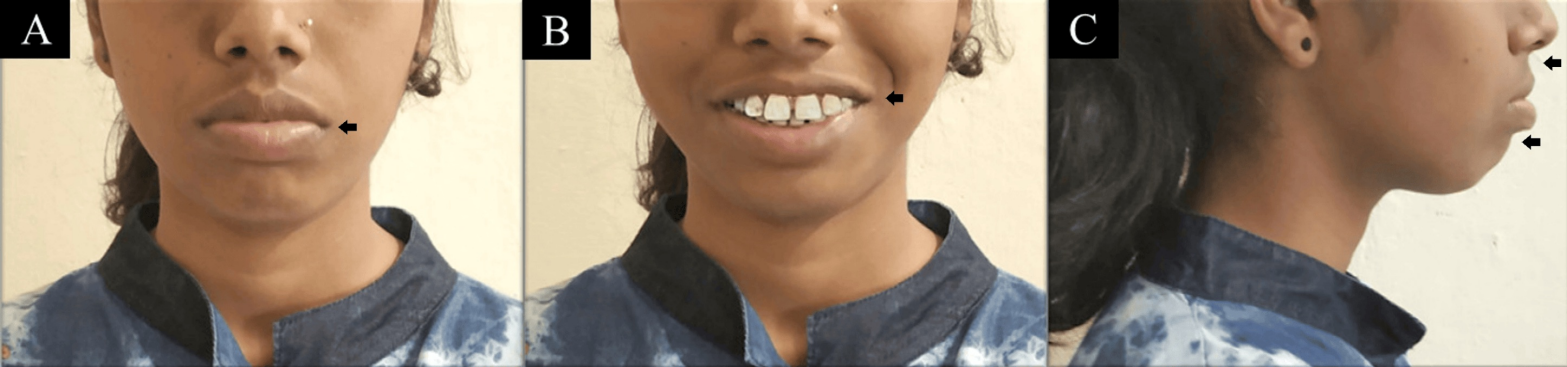
Extraoral pretreatment photographs: (A) frontal; (B) smiling; (C) profile.

A 22-year-old female patient came to the orthodontics department 18 months ago with the main complaint of forwardly positioned upper and lower front teeth. A convex profile was noticed during extraoral examination along with mesocephalic head form, mesoprosopic face form, acute nasolabial angle, deep mentolabial sulcus, potentially competent lips, and bilaterally symmetrical face [5]. The smile of the patient was symmetrical and consonant with full maxillary incisor display (Figure 1).

**Figure 2:**
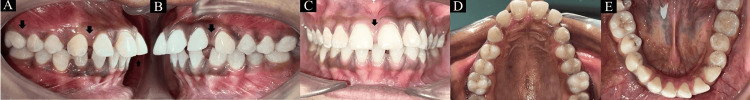
Intraoral pre-treatment photographs. (A) Right side view of buccal occlusion. (B) Left side view of buccal occlusion. (C) Frontal labial. (D) Occlusal surface of maxillary arch. (E) Occlusal surface of mandibular arch.

The intraoral examination revealed normal maxillary and mandibular alveolar ridge, normal tongue, normal labial and buccal vestibule, and healthy normal gingiva. Hard tissue examination revealed that all permanent teeth were present, with 5 mm overjet and 2 mm overbite. Both maxillary and mandibular anterior were proclined with the spacing in the anteriors of both the upper and lower arch. Both the right and left molar and canine relationship were class I (Figure 2).

**Figure 3:**
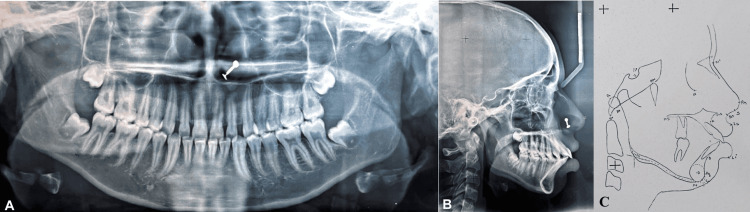
(A) Orthopantomogram. (B) Lateral cephalogram. (C) Tracing of lateral cephalogram.

An orthopantomogram examination showed that all of the teeth, including the third molars, were present in every quadrant. According to cephalometric analysis, the patient was in cervical vertebral maturation index (CVMI) stage VI (completion) with Class I skeletal bases and vertical growth patterns (SN-mandibular plane angle: 33°). She had proclined upper (1 to NA angle: 37°; 1 to NA mm: 11 mm) and lower incisors (1 to NB angle: 43°; 1 to NB mm: 12 mm). On soft tissue analysis, it was found that protrusive upper (4 mm prominence) and lower (3 mm prominence) lips. An acute nasolabial angle (94°) was found (Figure 3).

Treatment plan

The treatment objective was to correct the proclination of the upper and lower anterior to achieve normal overjet and overbite, to close pre-existing spacing, and to maintain the class I molar and canine relationship. Treatment started with the patient's motivation and education. The treatment plan includes bonding of both maxillary and mandibular arches and extraction of the first premolars of all four quadrants to correct proclination and prominence of lips. The alternative option can be the extraction of the second premolar [6]. After extraction, initial leveling and alignment were planned, followed by closure of extraction space using MV loop. To improve anchoring, the lingual arch in the mandible and the transpalatal arch in the maxilla were constructed.

Treatment progress

**Figure 4:**
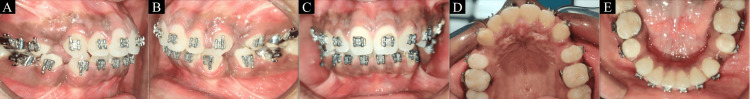
Intraoral stage photographs after initial leveling and alignment. (A) Right side view of buccal occlusion. (B) Left side view of buccal occlusion. (C) Frontal labial. (D) Occlusal surface of maxillary arch. (E) Occlusal surface of mandibular arch.

**Figure 5:**
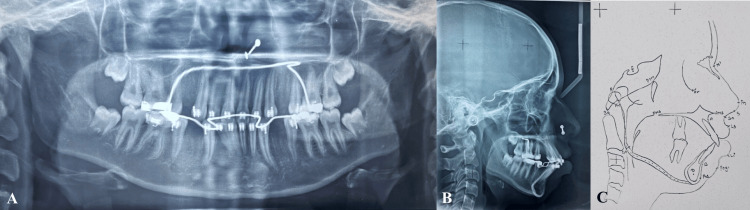
Stage radiographs: (A) Orthopantomogram; (B) lateral cephalogram; (C) tracing of lateral cephalogram.

The case was started by bonding both maxillary and mandibular arches using an MBT 0.022" slot prescription. After bonding, all quadrant's first premolars were extracted. The transpalatal arch and lingual arch were cemented in the maxillary and mandibular arch, respectively, to enhance anchorage. Initial leveling and alignment were practiced with a suitable arch wires sequence, which includes 0.016" NiTi wire, 0.016 × 0.022" NiTi wire, and 0.017 × 0.025" stainless steel (SS) wire [7] (Figure 4 and Figure 5).

**Figure 6:**
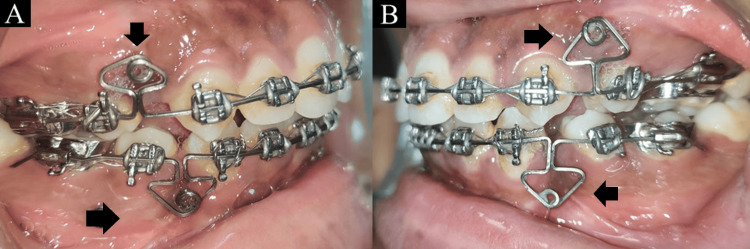
Intraoral records with multiple variability (MV) loop. (A) Right side view of buccal occlusion. (B) Left side view of buccal occlusion.

Arch wires were cinched distal to the molar to avoid further proclination of the anterior. An MV loop was used for demonstrating en masse retraction using 0.019 × 0.025" TMA wire. The 15° of alpha bend and 20° of beta bend are advised in order to create moments on the anterior and posterior teeth. The loop was activated by 2 mm every 1½ months with tight cinching back distal to molar (Figure 6).

**Figure 7:**
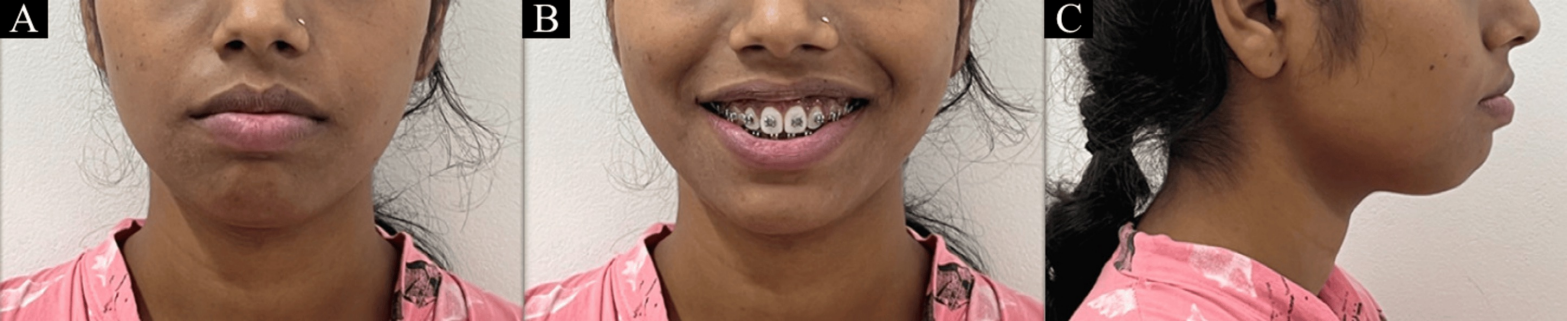
Extraoral current records. (A) Frontal view. (B) Smiling frontal view. (C) Profile view.

**Figure 8:**
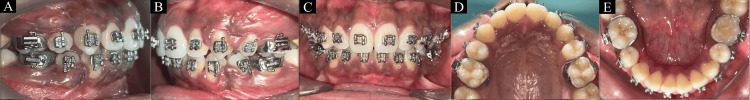
Intraoral current photographs. (A) Right side buccal occlusion. (B) Left side buccal occlusion. (C) Frontal view. (D) Maxillary occlusal view. (E) Mandibular occlusal view.

Retraction and complete extraction space closure were achieved within four months. Currently, both arches are well aligned with complete extraction space closure (Figure 7 and Figure 8).

**Figure 9:**
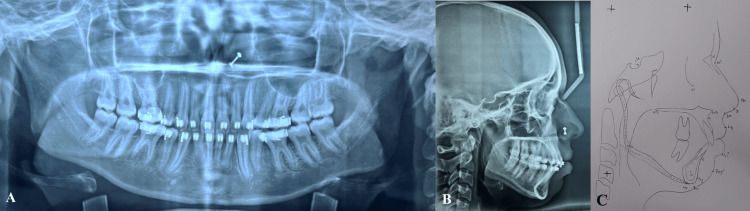
Current radiographs: (A) orthopantomogram; (B) lateral cephalogram; (C) tracing of lateral cephalogram.

At present, arch wires with finishing bends are given for finishing and detailing of the occlusion. To avoid relapse, the permanent lingual retainer is planned immediately after debonding [8].

## Discussion

Proclination refers to the forward inclination or tipping of teeth, typically in the anterior region, and plays a significant role in the development of malocclusions. Proclination of teeth affects both aesthetics and function. Orthodontic diagnosis and treatment planning should carefully consider proclination, aiming for optimal alignment, stability, and overall harmonious occlusion. To correct the proclination of anteriors, to achieve lip competency, and to decrease facial convexity, extraction of first premolars was carried out. Drobocky found that the patients who had their first premolars extracted experienced an average reduction in lip prominence of 3.4 and 3.6 mm with Ricketts’ E-line [9]. The closure of the extraction space must be taken into consideration in the treatment plan when the malocclusion is corrected by extracting the premolars.

In orthodontic procedure, it is essential to control tooth movement in all three planes (transverse, sagittal, and vertical) in order to achieve optimal tooth positions during the space closure. It is proven that maxillary and mandibular six anterior teeth can be retracted collectively or individually using the MV loop in an efficient manner that maintains optimal force. It measures 7 mm in the occluso-gingival height and 8 mm in the mesio-distal length. Because the loop has a lower vestibular height, it can adapt more readily in situations when the vestibule is shallow. It has the ability to regulate both vertical and horizontal tooth movement because of its nearly 8 mm horizontal and vertical arms. Constructing the MV loop takes only three minutes because of its simpler design. This method was used because the efficiency of this loop is equally good as that of the opus loop, with the additional advantage of comparatively easier wire bending. The MV loop's simplicity gives it a greater activation range and a lower breakage rate. In extraction cases, the MV loop shortens the total treatment duration [1].

When first premolar extraction is part of a treatment plan, a number of anchoring procedures are employed to retain anterior teeth [10]. Mini-implants and miniplates are examples of skeletal anchoring devices that are frequently utilized due to their increased comfort and improved aesthetics, but these types of anchoring devices are sometimes painful to patients, which is why they are not usually accepted by patients [11,12]. The lingual arch in the mandible and the transpalatal arch in the maxilla were selected in this clinical situation because these appliances are inexpensive, easy to fabricate, most reliable, and do not cause any discomfort to patients. The effectiveness of using these appliances appropriately and with effective anchor management was validated by the examination of entire and partial superimpositions.

## Conclusions

In conclusion, the application of the MV loop in the correction of protrusion of anteriors represents a promising and innovative approach in the field of orthodontics. This case report underscores the successful utilization of the MV loop technique, showcasing its efficacy in addressing complex malocclusions and achieving significant improvements in both functional and aesthetic aspects associated with malocclusion. It is important to note that individualized treatment plans, patient compliance, and long-term stability assessments remain crucial factors in the success of orthodontic interventions. As the field continues to evolve, further research and clinical studies are required to explore the broader applicability and long-term outcomes of the MV loop technique.
